# A Rare Presentation of Posterior Circulation Strokes: A Case Report of Nine Syndrome

**DOI:** 10.7759/cureus.68794

**Published:** 2024-09-06

**Authors:** Asham Al Salkhadi, Mohammad Ajwad Al Salkhadi, Mohammed B Baker

**Affiliations:** 1 Internal Medicine, Mubarak Al-Kabeer Hospital, Jabriya, KWT; 2 Faculty of Medicine, Jordan University of Science and Technology, Irbid, JOR; 3 Department of Ophthalmology, Faculty of Medicine, Jordan University of Science and Technology, Zarqa, JOR

**Keywords:** brainstem syndromes, case report, internuclear ophthalmoplegia, medial longitudinal fasciculus, nine syndrome, pontine paramedian reticular formation, pontine vascular syndromes

## Abstract

Nine syndrome is a rare variant of one-and-a-half syndrome that results from a stroke in the posterior circulation, mainly manifesting as ipsilateral horizontal gaze palsy, facial palsy, and contralateral hemiataxia. Awareness of the clinical signs of this syndrome is crucial for accurate localization of the lesion and guiding further investigations. We report a case of nine syndrome presenting with signs of one-and-a-half syndrome, ipsilateral facial nerve palsy, hemiataxia, and hemiparesis. There are only a few cases of nine syndrome reported in the literature.

## Introduction

Lesions affecting the brainstem can present with a wide range of clinical symptoms, including gaze palsies, due to the complexity of this area and the involvement of critical structures such as the medial longitudinal fasciculus (MLF), paramedian pontine reticular formation (PPRF), abducens nucleus, and adjacent facial colliculus [1]. The combination of unilateral horizontal conjugate gaze palsy and internuclear ophthalmoplegia (INO) in the other eye due to lesions in the MLF or PPRF was first described by Freeman in 1943 [2] and coined later as one-and-a-half syndrome by Miller Fisher in 1967 [3]. The combination of one-and-a-half syndrome with ipsilateral lower motor neuron facial nerve palsy constitutes the eight-and-a-half syndrome that was first described by Eggenberger in 1998 [4]. Moreover, when eight-and-a-half syndrome is associated with either hemiataxia or hemiparesis, it is termed nine syndrome, a rare variant of one-and-a-half syndrome that was reported for the first time by Rosini et al. [5].

The awareness of such a combination will help in the proper diagnosis of this syndrome and precise localization of the lesion, leading to more effective treatment. Here, we report a case of nine syndrome who presented with left-sided facial deviation and weakness of the right side of the body due to a brainstem stroke in the posterior circulation, along with its MRI findings and treatment plan.

## Case presentation

A 65-year-old man with a history of diabetes, hypertension, and a 15-pack-year smoking history presented to the emergency department with a one-day history of sudden dizziness, inability to close the right eye completely with left-sided mouth deviation, and right-sided weakness. He also reported binocular diplopia, left hemiataxia, and two episodes of non-bloody vomiting. There was no loss of consciousness, seizures, head trauma, or fever.

On examination, the patient had a Glasgow Coma Scale (GCS) score of 15/15 and stable vital signs. Neurological examination revealed decreased muscle power (3/5) and decreased sensation to light touch on the right side. Muscle tone was normal bilaterally with a negative Babinski sign. Cranial nerve examination showed left mouth deviation, left horizontal gaze palsy (impaired left eye abduction), and right eye adduction paresis, indicating internuclear ophthalmoplegia (INO). The remainder of the examination was unremarkable.

Laboratory investigations revealed an elevated HbA1c level of 8.9. Other laboratory results are shown in Table 1.

**Table 1:** Lab findings of the presented case Abbreviations: WBC, white blood cells; RBC, red blood cells; MCV, mean corpuscular volume; AST, aspartate transferase; ALT, alanine transferase; GGT, gamma glutamyl transferase; ALP, alkaline phosphatase; TSH, thyroid-stimulatig hormone; HDL, high-density lipoprotein; LDL, low-density lipoprotein

Lab test	Value	Normal range
Complete blood count		
WBC	12,000 cells/µL	4,500-11,000 cells/µL
RBC	5.7 million cells/µL	4.5-6 million cells/µL
Hemoglobin	16.9 g/dL	14-17 g/dL
MCV	86 fL	80-100 fL
Platelet count	220,000 cells/µL	150,000-450,000 cells/µL
Renal function test		
Glucose	12 mmol/L	3.9-5.6 mmol/L
Urea	5.7 mmol/L	2.5-7.1 mmol/L
Creatinine	71 µmol/L	61.9-114.9 µmol/L
Na	138 mEq/L	135-145 mEq/L
K	3.43 mEq/L	3.5-5.0 mEq/L
CO2	23 mEq/L	22-32 mEq/L
Protein	6.8 g/dL	6.0-8.0 g/dL
Albumin	3.8 g/dL	3.5-5.0 g/dL
Urate	3.49 mg/dL	2.5-7.0 mg/dL
Mg	1.67 mEq/L	1.5-2.5 mEq/L
Phosphorus	3.16 mg/dL	2.5-4.5 mg/dL
Ca	8.91 mg/dL	8.5-10.5 mg/dL
Liver function test		
AST	17 IU/L	0-40 IU/L
ALT	16 IU/L	0-40 IU/L
GGT	26 IU/L	0-55 IU/L
ALP	70 IU/L	40-120 IU/L
Total bilirubin	10.2 µmol/L	5.1-17 µmol/L
Direct bilirubin	2 µmol/L	1.7-5.1 µmol/L
Thyroid function test		
TSH	0.71 mIU/L	0.4-4.5 mIU/L
T4	14.8 µg/dL	4.5-11.5 µg/dL
Lipid profile		
Total cholesterol	4.1 mmol/L	<5.2 mmol/L
Triglyceride	2.47 mmol/L	<1.7 mmol
HDL	1 mmol/L	>1.0 mmol/L
LDL	1.97 mmol/L	<3.0 mmol/L

**Figure 1:**
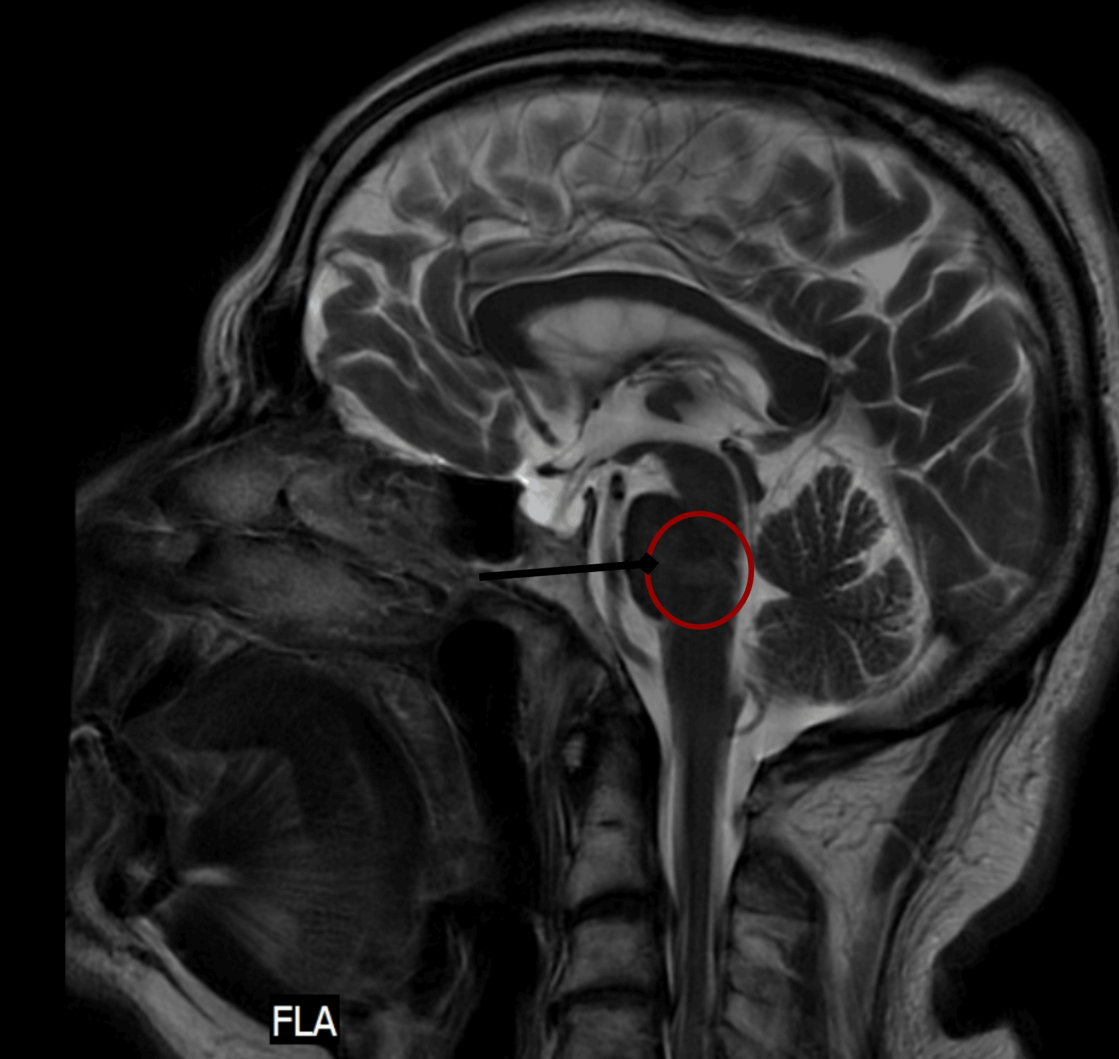
Sagittal view of T2 brain MRI revealed para-sagittal, non-enhancing, restricted hyperintense focus in the posterior aspect of the left side of the pons, indicating the early stage of a subacute infarct MRI: magnetic resonance imaging

**Figure 2:**
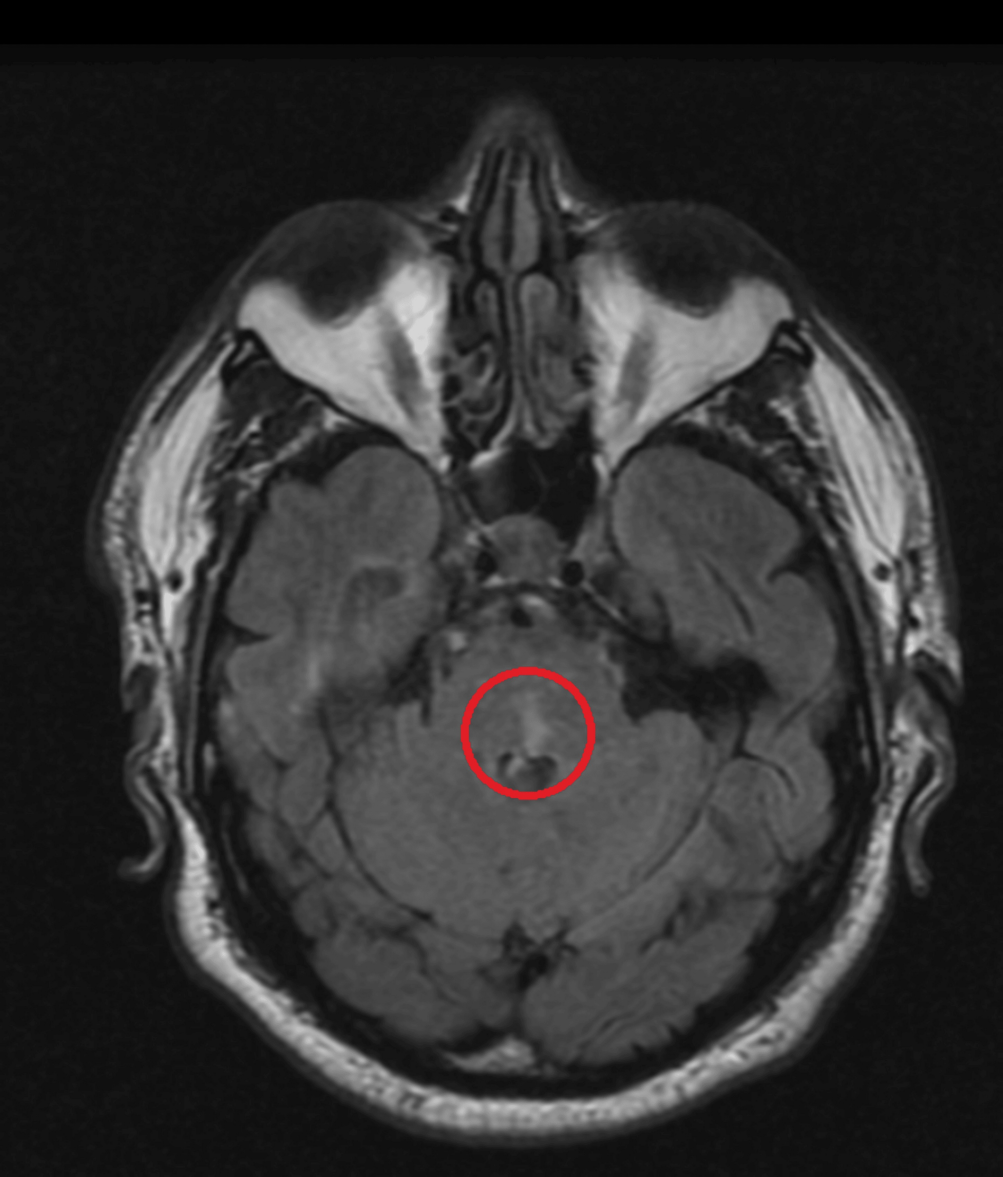
Axial view of T2 brain MRI revealed para-sagittal, non-enhancing, restricted hyperintense focus in the posterior aspect of the left side of the pons, indicating the early stage of a subacute infarct MRI: magnetic resonance imaging

A non-contrast CT scan of the brain showed no evidence of Intra- or extra-axial brain hemorrhage. MRI revealed a para-sagittal, non-enhancing, restricted hyperintense focus in the posterior aspect of the left side of the pons, likely indicating the early stage of a subacute infarct (Figures 1, 2).

Echocardiography showed a normal left ventricular size, filling pressure, and systolic function, with no regional wall motion or valvular abnormalities. The patient was diagnosed with a left-sided pontine ischemic stroke but was ineligible for tPA treatment due to presentation outside the thrombolysis time window.

However, his management plan focused on risk factor control, including blood pressure regulation with lisinopril 20 mg, lipid control with high-intensity statin, and antiplatelet therapy with aspirin 75 mg to reduce the risk of recurrent stroke. Moreover, strict glycemic control was prioritized, with metformin prescribed as part of the glucose management to maintain optimal blood sugar levels. The plan emphasized lifestyle modifications, such as a balanced diet, smoking cessation, and regular physical activity. In addition, he was enrolled in a physiotherapy program. The patient's limb weakness has partially improved, with total resolution of ocular symptoms. Regular follow-up appointments were scheduled to monitor the patient’s progress, glycemic control, and overall response to the treatment plan.

## Discussion

Horizontal eye movement is coordinated by several brainstem structures, including the PPRF, the Abducens nucleus, and the MLF. The PPRF is responsible for generating horizontal saccades [6], the Abducens nucleus controls the lateral rectus muscle of the eye, and the MLF connects the abducens nucleus (which controls lateral eye movement) to the contralateral oculomotor nucleus (which controls medial eye movement), allowing horizontal conjugate lateral gaze and saccadic eye movements. Disorders of horizontal eye movement often result from damage to these structures, leading to various syndromes with distinct clinical features.

One-and-a-half syndrome is a rare neurological condition characterized by a combination of one ipsilateral conjugate horizontal gaze palsy and one-half of an ipsilateral INO [7]. In this syndrome, patients cannot move the affected eye horizontally in either direction, while the contralateral eye can only move laterally. However, vertical eye movements and convergence are preserved. Our patient showed these signs. This condition is caused by a lesion in the pons, affecting both the PPRF (or the Abducens nucleus) and the MLF. While demyelinating diseases such as multiple sclerosis or neoplastic lesions can cause one-and-a-half syndrome, vascular lesions, particularly strokes, are the most common cause [8]. In our case, the patient had a stroke.

A notable combination of symptoms was described by Eric Eggenberger in 1998, where one-and-a-half syndrome was associated with ipsilateral lower motor neuron facial nerve (CN VII) palsy. This unique presentation, observed in our patient and referred to as eight-and-a-half syndrome, results from a lesion that extends to involve the facial nerve nucleus in addition to the structures associated with one-and-a-half syndrome (1½ + 7).

An even rarer variant, known as nine syndrome, occurs when eight-and-a-half syndrome is accompanied by either hemiataxia or hemiparesis. In this case, the patient had both hemiataxia and hemiparesis leading to a diagnosis of nine syndrome. A lesion in the corticospinal tract or medial lemniscus can result in a hemiparesis/hemianesthesia variant, while a lesion in the inferior cerebellar peduncle or red nucleus in the midbrain leads to the ataxic variant [9]. This condition, which was first reported by Rosini et al., highlights the complex interplay between the motor pathways of the face, eyes, and limbs within the brainstem. These cases are significant because they illustrate how extensive pontine lesions can affect multiple cranial nerve functions and motor pathways.

Understanding these syndromes is crucial for accurate diagnosis and management. Prompt recognition of the underlying causes, such as vascular lesions, is essential for effective treatment and can impact patient outcomes. Advanced imaging techniques, including MRI, are instrumental in identifying the precise location and extent of brainstem lesions, guiding appropriate therapeutic interventions [10].

In conclusion, the intricate anatomy and physiology of the brainstem play a pivotal role in coordinating eye movements and facial expressions. Damage to specific areas within this region can lead to distinct and sometimes overlapping neurological syndromes, emphasizing the need for a thorough understanding of these pathways in clinical practice.

## Conclusions

Nine syndrome is an advanced form of one-and-a-half syndrome that affects additional structures in the midbrain and medulla. Recognizing the symptoms of nine syndrome early is crucial for identifying the affected brain regions and potential causes. Early recognition facilitates immediate treatment, which can significantly improve patient outcomes while waiting for diagnostic test results. Symptoms typically include unilateral horizontal gaze palsy, internuclear ophthalmoplegia, lower motor neuron facial nerve palsy, and either hemiataxia or hemiparesis. Well-timed diagnosis and intervention are essential for optimizing recovery and minimizing complications.
